# Zur Publikationsleistung der universitären Unfallchirurgie in Deutschland

**DOI:** 10.1007/s00104-021-01538-y

**Published:** 2021-11-30

**Authors:** J. Preut, K.-H. Frosch, E. S. Debus, R. T. Grundmann

**Affiliations:** 1 Klinik und Poliklinik für Gefäßmedizin, Universitäres Herzzentrum, Universitätsklinikum Hamburg-Eppendorf, Hamburg, Deutschland; 2 Klinik und Poliklinik für Unfallchirurgie und Orthopädie, Universitätsklinikum Hamburg-Eppendorf, Hamburg, Deutschland; 3 Berufsgenossenschaftliches Klinikum Hamburg, Hamburg, Deutschland

**Keywords:** Universitätskliniken, Publikationsrate, Forschungsaktivität, Benchmark, Journal-Impact-Faktor, University hospitals, Publication rate, Research activity, Benchmark, Journal impact factor

## Abstract

**Hintergrund:**

Zu den Leistungskriterien einer Universitätsklinik gehören ihre Publikationsaktivitäten. Ziel der vorliegenden bibliometrischen Untersuchung war es, die Publikationsaktivitäten deutscher unfallchirurgischer Universitätskliniken in einem Benchmarking vergleichend darzustellen.

**Material und Methodik:**

Die Publikationsleistung der Führungsmannschaften, bestehend aus Chef- und Oberärzten, Sektions- und Bereichsleitern von 39 deutschen unfallchirurgischen Universitätskliniken wurde über 10 Jahre (01.01.2010 bis 31.12.2019) erfasst. Berücksichtigt wurden alle Publikationen, die in PubMed gelistet waren und bei denen die entsprechenden Personen Erst- oder Letztautor waren. Zusätzlich wurde der Impact-Faktor (IF) bestimmt.

**Ergebnisse:**

Insgesamt wurden 4438 Veröffentlichungen erfasst, publiziert von 381 Chirurgen. Der Anteil der publizierenden Mitarbeiter betrug 72,8 %. Publiziert wurde in 545 Journalen. Der durchschnittliche IF aller Publikationen war 1,81. Die Publikationsaktivitäten der Kliniken zeigten eine hohe Streubreite, dies galt sowohl für die Publikationsanzahl als auch für die generierten IF des einzelnen Mitarbeiters. Die Publikationsaktivität reichte von durchschnittlich 16,4 Publikationen pro Mitarbeiter in der bestplatzierten Klinik bis 1,5 Publikationen bei der letztplatzierten. Gleiches ergab die Summe der IF. In der nach diesem Maßstab bestplatzierten Klinik erzielte der einzelne Mitarbeiter durchschnittlich kumuliert 42,1 IF verglichen mit 1,7 IF bei der letztplatzierten.

**Schlussfolgerung:**

Die Publikationsleistung deutscher unfallchirurgischer Universitätskliniken zeigt eine hohe Varianz, wie dies auch bei anderen Disziplinen gefunden wurde. Die Ursachen müssen offen bleiben, eine unterschiedliche Forschungsmotivation ist aber nicht auszuschließen.

## Abstract (en)

**Background:**

One of the performance criteria of a university hospital is its publication activities. The aim of this bibliometric study was a comparative benchmarking of the publication activities of German orthopedic trauma surgery university hospitals.

**Material and methods:**

The publication performance of the leading groups, consisting of chief and senior physicians, section and division heads of 39 German orthopedic trauma surgery university hospitals, was recorded over a period of 10 years (1 January 2010–31 December 2019). All publications that were listed in PubMed and for which the appropriate persons were first or last author were considered. In addition, the impact factor (IF) was determined.

**Results:**

A total of 4438 publications were recorded published by 381 surgeons. The share of publishing authors was 72.8%. The articles were published in 545 journals. The average IF of all publications was 1.81. The publication activities of hospitals showed a wide range, this applied to both the number of publications and the IF generated by the individual author. The publication activity ranged from an average of 16.4 publications per author in the top-ranked hospital to 1.5 publications in the last-placed hospital. The same result was seen with the total IFs. In the highest ranking hospital according to this criterion the individual surgeon achieved on average of 42.1 cumulative IFs compared with 1.7 IFs in the last placed hospital.

**Conclusion:**

The publication performance of German orthopedic trauma surgery university hospitals showed a high variance, as was also found in other disciplines. The causes must remain open, but a different research motivation cannot be ruled out.

## Hintergrund

Zur Publikationsaktivität deutscher Universitätskliniken gibt es nur wenige Daten. Putzer et al. [11] verglichen die Publikationsleistung der Universitätskliniken für Anästhesiologie in Deutschland, Österreich und der Schweiz in den Jahren 2001 bis 2010. Sie demonstrierten im Benchmarking erhebliche Unterschiede zwischen den Kliniken in der Anzahl an Publikationen und den dabei erzielten Impact-Faktoren (IF). Große Universitätskliniken führten die Rangliste an. Auf die Anzahl an Mitarbeitern einer Klinik wurden die Daten allerdings nicht bezogen, sodass über die durchschnittliche Aktivität des einzelnen Mitarbeiters (Publikationsleistung pro Personaleinheit) keine Aussage gemacht werden konnte.

Schwarzer et al. [13] berücksichtigten dies bei einem Vergleich der Publikationsaktivitäten deutscher Universitätskliniken der Herzchirurgie, Kardiologie und Allgemeinchirurgie der Jahre 2011 bis 2013. Sie stellten fest, dass sich die Publikationsleistung gemessen an der Anzahl an Publikationen zwischen den einzelnen Fächern nicht signifikant unterschied, wenn die Zahl der Mitarbeiter berücksichtigt wurde. Allerdings war der durchschnittliche IF der kardiologischen Publikationen deutlich höher als in den chirurgischen Fächern, was die Autoren auf die größere Verbreitung kardiologischer Zeitschriften zurückführten.

Ein Benchmarking einzelner Kliniken lieferten Schwarzer et al. nicht. Dies machten für die chirurgischen Fächer erstmals Schubert et al. [12], die erhebliche Unterschiede in der Publikationsaktivität der Führungsmannschaft (Chef, Oberärzte und Abteilungsleiter) in der plastischen Chirurgie deutscher Universitätsklinken beobachteten, je nachdem, ob es sich um eigenständige Kliniken oder untergeordnete Abteilungen und Strukturen handelte. Selbstständige Einheiten waren signifikant aktiver. Nach der gleichen Methodik gingen Haffke et al. [7] und Debus et al. [2] vor, die ebenfalls demonstrierten, dass eigenständige Abteilungen in der Gefäßchirurgie sehr viel mehr Publikationen aufwiesen als nichtselbstständige Organisationsstrukturen.

Debus et al. [3] veröffentlichten auch ein Benchmarking der Führungsmannschaften sämtlicher deutscher herzchirurgischer Universitätskliniken in einem 10-Jahres-Zeitraum und stellten gravierende Unterschiede fest: Anonymisiert publizierte das aktivste Quintil aller Kliniken 39 % aller Publikationen, das am wenigsten aktive Quintil nur 9,4 %. Bezogen auf den einzelnen Mitarbeiter, brachten es die Mitarbeiter des aktivsten Klinikquintils durchschnittlich auf 11,9 Publikationen (29,6 IF), die Mitarbeiter des publikationsschwächsten Quintils hingegen nur auf 3,3 Publikationen (8,0 IF) als Erst- oder Letztautoren im genannten Zeitraum.

Ob diese erheblichen Diskrepanzen sich bei den Publikationsaktivitäten deutscher Universitätskliniken für Unfallchirurgie resp. Unfallchirurgie/Orthopädie in gleicher Weise beobachten ließen, sollte in der vorliegenden Untersuchung überprüft werden. Zusätzlich wurde untersucht, in welchen Zeitschriften bevorzugt veröffentlicht wurde.

## Material und Methodik

Erfasst wurde die Publikationsleistung der Führungsmannschaften, bestehend aus Chef- und Oberärzten (nicht Funktionsoberärzten), Sektions- und Bereichsleitern von 39 deutschen unfallchirurgischen Universitätskliniken. Bei 19 Kliniken handelte es sich ausschließlich um Lehrstühle für Unfallchirurgie, bei 20 Kliniken um solche für Unfallchirurgie und Orthopädie. Die Personalbesetzung wurde den Webseiten der Klinken entnommen. Stichtag aller Betrachtungen (Personalbesetzung und Publikationen) war der 01.01.2020. Der Beobachtungszeitraum erstreckte sich über 10 Jahre vom 01.01.2010 bis 31.12.2019. Berücksichtigt wurden alle Publikationen, die in PubMed (Medline) gelistet waren, zum Stichtag in gedruckter Form vorlagen und bei denen es sich um Originalarbeiten mit Abstract handelte, Kommentare und Antworten entfielen. Für jedes Mitglied der Führungsmannschaft wurden nach diesen Kriterien sämtliche Publikationen (Titel der Publikation, Monat und Jahr der Veröffentlichung sowie Journal der Veröffentlichung) ermittelt, bei denen die entsprechenden Personen entweder Erst- oder Letztautor waren. Bei Arbeitsplatzwechsel des Autors im 10-Jahres-Zeitraum wurde die Publikationsleistung des Autors der Klinik zugeordnet, an der der Autor zum Stichtag beschäftigt war.

In einem zweiten Schritt wurde für jedes erfasste Journal der 5‑Jahres-Impact-Faktor des Jahres 2016 (5-Jahres-IF 2016) in „Web of Science“ bestimmt. Hatte das Journal keinen 5‑Jahres-IF 2016 aufzuweisen, wurde der Impact-Faktor (IF) des Publikationsjahres verwendet. Journale ohne IF gingen zwangsläufig nur in die Berechnung der Anzahl an Publikationen ein. Von Zeitschriften vergebene Eigenfaktoren wurden nicht gewertet.

Um die Aussagekraft des IF abschätzen zu können, wurde für alle Journale zusätzlich mit Stichtag 01.03.2021 der h‑Index des jeweiligen Journals in der Datenbank des SCImago Journal & Country Rank [14] bestimmt. Der kumulierte h‑Index des einzelnen Mitarbeiters ergab sich analog zu den kumulierten IF durch die Gesamtsumme der h‑Indizes der jeweiligen Journale, in denen die Publikationen veröffentlicht wurden.

Für die Berechnung der Gesamtsumme an Publikationen einer Klinik, die Gesamtsumme an Impact-Faktoren einer Klinik, für die Journalanalyse und die Analyse der Publikationsschwerpunkte wurden in einem zweiten Schritt Doppelpublikationen entfernt. Eine sog. Doppelpublikation lag vor, wenn ein Mitglied der Führungsmannschaft Erst- und ein anderes Letztautor derselben Publikation einer Klinik waren. Doppelberechnungen einzelner Publikationen finden sich demnach in dem vorliegenden Klinikranking nicht. Lediglich bei der Publikation einer Multicenterstudie wurde diese beiden beteiligten Kliniken zugerechnet. Dies betraf 171 Publikationen.

### Klinikranking

Da die Kliniken eine unterschiedliche Mitarbeiteranzahl hatten, erfolgte das Klinikranking (Platz 1–39) anhand der Zahl der Publikationen pro Mitarbeiter. Zusätzlich wurden die Anzahl der Publikationen pro publizierendem Mitarbeiter und die Gesamtzahl der Publikationen einer Klinik erfasst. Analog wurden IF pro Chirurg, IF pro publizierendem Chirurg und die Gesamtzahl der IF einer Klinik bestimmt. Das Ranking kann folglich analog der Publikationsanzahl auch nach diesen Parametern individuell bestimmt werden und ist so dem Urteil des Betrachters überlassen.

Um die Unterschiede zwischen den Klinken noch deutlicher zu machen, wurden in einem weiteren Schritt die Kliniken anhand der Publikationen pro Chirurg in Quartile (Q) eingeteilt, Q1 (*n* = 10), Q2 (*n* = 10), Q3 (*n* = 9), Q4 (*n* = 10), mit Q1 mit den wenigsten und Q4 mit den meisten Publikationen pro Chirurg. Diese Einteilung der Kliniken wurde beibehalten und zusätzlich überprüft, wie sich die Kliniken von Q1 bis Q4 in ihren Publikationen pro publizierendem Chirurg, ihren IF pro Chirurg und ihren IF pro publizierendem Chirurg unterschieden.

### Statistische Auswertung

Zur statistischen Auswertung wurden die Mittelwerte mittels einfaktorieller Varianzanalyse verglichen. Zusätzlich wurden alle einzelnen Gruppen per t‑Test verglichen. Als Signifikanzniveau wurde *p* < 0,05 gewählt.

## Ergebnisse

### Publikationsanalyse

Insgesamt wurden 4438 Veröffentlichungen erfasst, publiziert von 381 Chirurgen (bei insgesamt 523 Mitarbeitern). Der Anteil der publizierenden Mitarbeiter betrug demnach insgesamt 72,8 %. 120 (31,5 %) Autoren waren Professoren, 109 (28,6 %) Privatdozenten, 147 (38,6 %) Doktoren und 5 (1,3 %) der Autoren hatten keinen Titel. In der Summe veröffentlichten Professoren als Erst- oder Letztautor 2646 Publikationen (21,9/Mitarbeiter), Privatdozenten 1586 (14,4/Mitarbeiter), Doktoren 789 (3,0/Mitarbeiter) und Mitarbeiter ohne Titel 10 Publikationen (0,3/Mitarbeiter), Doppelpublikationen eingerechnet. Bei den Erstautorenschaften führten die Privatdozenten mit einem Anteil von 41 % vor den Professoren mit 34 %, bei den Letztautorenschaften die Professoren mit 69,6 % vor den Privatdozenten mit 22,9 %.

Publiziert wurde in 545 Journalen, wobei *Der Unfallchirurg* mit 404 Publikationen (9,1 %) und *Zeitschrift für Orthopädie und Unfallchirurgie* mit 253 (5,7 %) Publikationen führend waren. Tab. 1 nennt die 20 Zeitschriften, in denen am häufigsten publiziert wurde und die Summe ihrer kumulierten Impact-Faktoren.

**Table 1:** 

Rang	Journal	Publikationen	Anteil (%)	5‑Jahres-IF	CIF	Anteil CIF (%)
1	*Unfallchirurg*	404	9,1	0,631	255,0	3,2
2	*Z Orthop Unfall*	253	5,7	0,595	150,5	1,9
3	*Arch Orthop Trauma Surg*	228	5,1	1,905	434,3	5,4
4	*Injury*	206	4,6	2,409	496,3	6,2
5	*Orthopade*	200	4,5	0,561	112,1	1,4
6	*Int Orthop*	160	3,6	2,631	421,0	5,3
7	*BMC Musculoskelet Disord*	115	2,6	2,268	260,8	3,3
8	*Eur J Trauma Emerg Surg*	104	2,3	1,123	116,7	1,5
9	*Knee Surg Sports Traumatol Arthrosc*	102	2,3	3,284	335,0	4,2
10	*Oper Orthop Traumatol*	92	2,1	0,778	71,5	0,9
11	*Eur Spine J*	71	1,6	2,937	208,5	2,6
12	*Chirurg*	63	1,4	0,622	39,2	0,5
13	*Technol Health Care*	54	1,2	0,615	33,2	0,4
14	*J Shoulder Elbow Surg*	52	1,2	3,089	160,6	2,0
15	*J Orthop Res*	45	1,0	2,982	134,2	1,7
15	*PLoS One*	45	1,0	3,394	152,7	1,9
17	*Acta Chir Orthop Traumatol Cech*	44	1,0	0,346	15,2	0,2
18	*J Orthop Trauma*	35	0,8	2,264	79,2	1,0
18	*Clin Orthop Relat Res*	35	0,8	3,907	136,7	1,7
18	*Acta Orthop Belg*	35	0,8	0,455	15,9	0,2
*Summe*	*n* *=* *20*	*2343*	*52,8*	*MW* *=* *1,84*	*3628,8*	*45,3*
*Andere*	*n* *=* *525*	*2095*	*47,2*	*MW* *=* *2,20*	*4380,2*	*54,7*
*Gesamt*	*n* *=* *545*	*4438*	*100*	*MW* *=* *2,18*	*8009,0*	*100*

*CIF* kumulierter Impact-Faktor,* IF* Impact-Faktor, *MW* Mittelwert

Der durchschnittliche IF aller Publikationen war 1,81. Je höher der Impact-Faktor einer Zeitschrift war, desto geringer war ihr Anteil an der Gesamtzahl der Publikationen: Publikationen mit einem IF <1 machten 39,2 % (*n* = 1737) aller 4438 Veröffentlichungen aus, solche mit IF ≥1 bis <2 18,5 % (*n* = 822), Publikationen mit ≥2 bis <3 24,7 % (*n* = 1096), mit ≥3 bis <4 11,8 % (*n* = 523) und Publikationen mit einem IF ≥4 5,9 % (*n* = 260).

Insgesamt 54,2 % (*n* = 2405) der Publikationen fokussierten auf Traumatologie im engeren Sinn, 22,2 % (*n* = 984) auf Orthopädie, 15,7 % (*n* = 695) auf Orthopädie und Traumatologie, 2,8 % (*n* = 124) auf Handchirurgie, 1,7 % (*n* = 75) auf Sportmedizin, 1,4 % (*n* = 60) auf plastische Chirurgie und 2,1 % (*n* = 95) auf interdisziplinäre Themen.

Mit den 4438 Publikationen wurden 8009 kumulierte Impact-Faktoren (IF) erzielt.

### Klinikranking

Das Ranking der einzelnen Kliniken ist in Tab. 2 aufgeführt. Die Reihenfolge der Kliniken erfolgte dabei nach der Zahl der Publikationen pro Mitarbeiter. Die erstplatzierte Abteilung weist 16,4 Publikationen pro Mitarbeiter auf, die letztplatzierte 1,5 Publikationen. Diese Reihenfolge entsprach weitgehend dem Ranking anhand der Zahl der Publikationen pro publizierendem Mitarbeiter. Eine weitere Möglichkeit ist ein Ranking nach dem kumulierten IF einer Klinik oder dem IF pro Mitarbeiter der jeweiligen Klinik. So nahm nach den pro Chirurg erzielten IF die Klinik 2 mit 42,1 IF den ersten, die Klinik 4 mit 31,3 IF den zweiten und die Klinik 9 mit 23,8 IF den dritten Platz ein.

**Figure 1:**
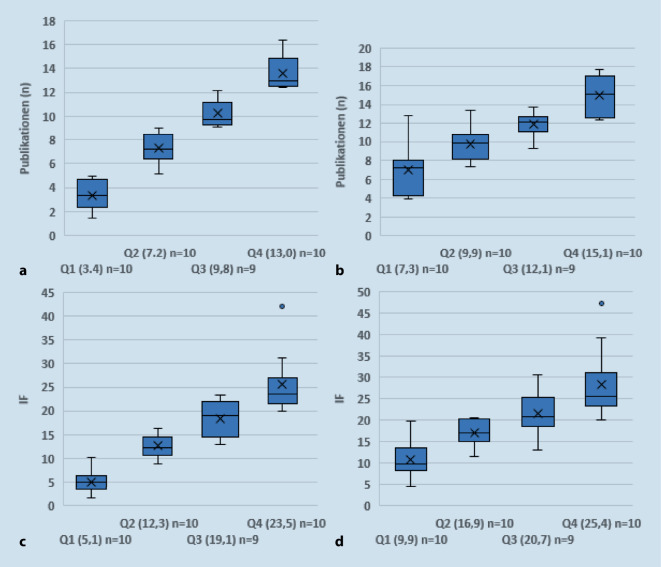

Ganz gleich, nach welchem Kriterium das Ranking erfolgt, entscheidend sind die gravierenden Unterschiede zwischen den Kliniken. Dies macht Abb. 1 deutlich, in der die Publikationsaktivitäten der einzelnen Klinikquartile aufgeführt sind. Stets befinden sich die Kliniken der Quartile 1, 2, 3 und 4 (Ranking nach Publikationen pro Mitarbeiter) im gleichen Quartil, ob nun nach Publikationen pro publizierendem Mitarbeiter, IF pro Mitarbeiter oder IF pro publizierendem Mitarbeiter unterschieden wird. Das publikationsaktivste Quartil (Q4) – es machte 40,1 % aller Publikationen aus – wies im Median 13,0, das publikationsschwächste (Q1) – sein Anteil an allen Publikationen betrug 11,5 % –3,4 Publikationen pro Mitarbeiter auf, die Zahl an Publikationen pro publizierendem Mitarbeiter war in Q4 15,1, in Q1 7,3. Die kumulierten IF pro Mitarbeiter betrugen im Median in Q4 23,5, in Q1 5,1 und die kumulierten IF pro publizierendem Mitarbeiter waren in Q4 25,4 vs. 9,9 in Q1.

**Table 2:** 

Rang	Publ. pro MA	IF pro MA	Publ. gesamt	IF gesamt	MA (publ. MA)	
*1*	16,4	22,5	213	292,2	13	(12)
*2*	15,1	42,1	136	378,8	9	(8)
*3*	14,7	25,3	147	253,3	10	(9)
*4*	13,8	31,3	345	782,3	25	(20)
*5*	13,0	21,4	208	341,6	16	(13)
*6*	12,9	25,4	142	279,5	11	(10)
*7*	12,7	21,5	89	150,8	7	(7)
*8*	12,6	23,1	226	416,5	18	(17)
*9*	12,4	23,8	149	285,2	12	(12)
*10*	12,4	20,0	124	200,5	10	(10)
*11*	12,2	21,5	158	280,0	13	(13)
*12*	11,7	19,3	175	289,2	15	(14)
*13*	10,7	19,1	107	190,8	10	(10)
*14*	10,6	22,3	254	535,6	24	(22)
*15*	9,8	13,7	205	287,8	21	(16)
*16*	9,4	15,6	85	140,0	9	(7)
*17*	9,3	13,0	56	77,8	6	(6)
*18*	9,2	23,4	120	304,4	13	(10)
*19*	9,1	17,3	137	260,2	15	(10)
*20*	9,0	13,9	72	111,5	8	(7)
*21*	8,9	12,8	160	229,7	18	(15)
*22*	8,3	16,2	83	161,7	10	(8)
*23*	8,0	10,8	40	53,8	5	(3)
*24*	7,6	14,0	99	181,8	15	(11)
*25*	6,8	10,6	81	126,7	12	(11)
*26*	6,5	11,4	110	194,4	17	(12)
*27*	6,4	16,4	32	81,8	5	(4)
*28*	6,3	11,8	132	247,1	21	(14)
*29*	5,1	8,8	41	70,7	8	(5)
*30*	5,0	6,6	50	66,4	10	(7)
*31*	4,7	5,9	52	65,1	11	(6)
*31*	4,7	10,2	52	112,0	11	(7)
*33*	3,9	5,9	193	297,2	50	(15)
*34*	3,4	4,2	31	37,8	9	(8)
*35*	3,3	6,3	59	113,3	18	(9)
*36*	3,0	3,8	15	19,1	5	(2)
*37*	2,6	3,3	31	40,0	12	(4)
*38*	1,6	1,7	13	13,5	8	(3)
*39*	1,5	3,5	16	38,9	13	(4)
*Summe*	*–*	*–*	*4438*	*8009*	*523*	*(381)*

*IF* Impact-Faktor, *MA* Mitarbeiter, *Publ.* Publikationen, *publ. *publizierende

^a^Die Nummerierung der Kliniken folgt der durchschnittlichen Anzahl der Publikationen des einzelnen Mitarbeiters

**Figure 2:**
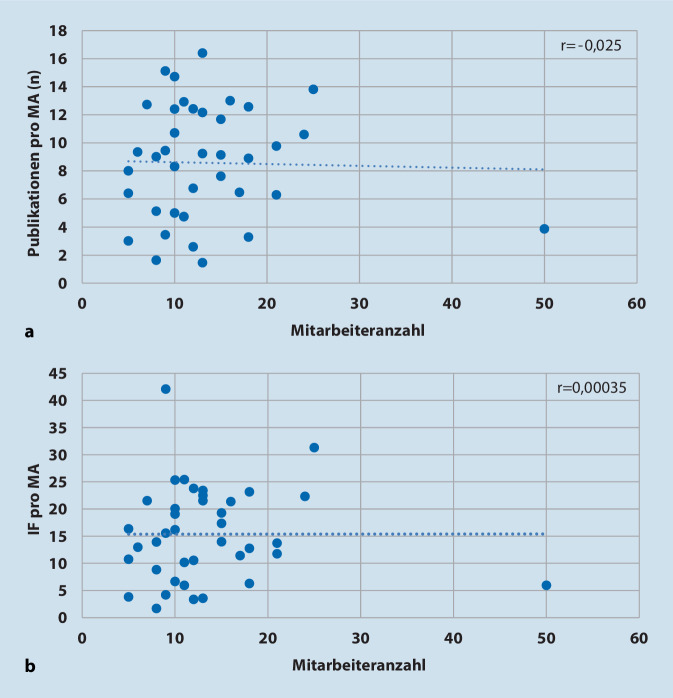

Wie in Abb. 2 demonstriert, bestand zwischen der Zahl der Mitarbeiter und der Anzahl an Publikationen pro Mitarbeiter keine Korrelation. Ebenso wenig gab es eine Korrelation zwischen Mitarbeiteranzahl und IF pro Mitarbeiter.

**Figure 3:**
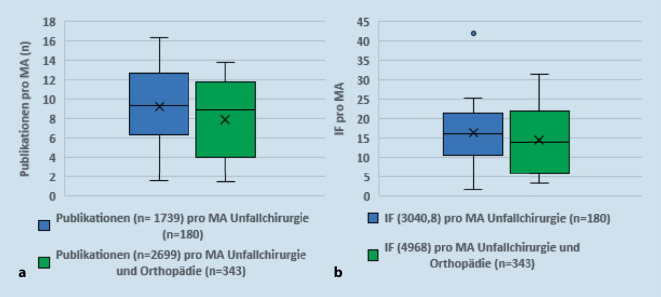

Da in der vorliegenden Untersuchung ein Klinikranking der Unfallchirurgie erfolgte, aber bei der Hälfte der Klinken Unfallchirurgie und Orthopädie zusammengeführt waren, wurde auch nach der Klinikstruktur unterschieden. Wie Abb. 3 zeigt, unterschieden sich rein unfallchirurgische und unfallchirurgisch/orthopädische Kliniken nicht signifikant in der Publikationsleistung des einzelnen Mitarbeiters. Die Zahl der Publikationen pro Mitarbeiter betrug im Median bei der Unfallchirurgie 9,3 (1,6–16,4), bei Unfallchirurgie/Orthopädie 8,9 (1,5–13,8; *p* = 0,308). Die Summe der IF pro Mitarbeiter machte in der Unfallchirurgie im Median 16,2 (1,7–42,7), in der Unfallchirurgie/Orthopädie 13,8 (3,3–31,3; *p* = 0,519) aus.

**Figure 4:**
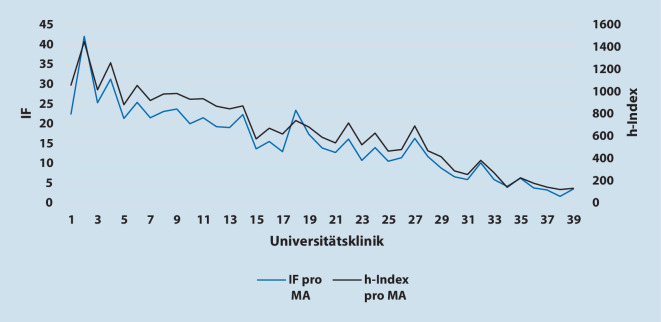

Um zu überprüfen, ob der IF ein adäquates Maß für ein Benchmarking darstellt, wurde zusätzlich der h‑Index pro Mitarbeiter bestimmt. Abb. 4 demonstriert die identische Aussage im Ranking nach Summe der IF pro Mitarbeiter und h‑Indizes pro Mitarbeiter. Die Klinik mit dem höchsten IF/Mitarbeiter (Klinik 2, Tab. 2, mit 42,1 IF) hatte auch den höchsten h‑Index (1449), die Klinik mit dem geringsten IF (Klinik 38, Tab. 2, mit 1,7 IF) den geringsten h‑Index (122) pro Mitarbeiter.

## Diskussion

Im Jahr 2011 veröffentlichten Gebhard et al. [5] einen aufschlussreichen Artikel über das universitäre Benchmarking für die Unfallchirurgie in Deutschland. Sie kamen zu dem Schluss, dass die Summe der zugewiesenen Geldmittel in keiner Weise Rückschlüsse auf die zugrunde liegende Forschungsleistung oder Lehrleistung der entsprechenden Abteilung erlaubt. Die Summe der eingeworbenen Drittmittel korrelierte auch nicht mit der Anzahl der daraus entstandenen Publikationen oder der erreichten Impact-Punkte. Sie folgerten, dass das einzige Kriterium, das es ermöglicht, die Leistungen im Bereich der Forschung als Kriterium für ein interuniversitäres Ranking zugrunde zu legen, nach sorgfältigem Durchgehen aller vorhandenen Daten der mittlere Impact-Faktor je Publikation sei. Genau dieser Ansatz ist nun hier erstmals vergleichend für die Führungsmannschaften aller unfallchirurgischen Universitätskliniken Deutschlands über einen 10-Jahres-Zeitraum durchgeführt worden, wobei sich die Auswahl der Publikationen auf Erst- und Letztautorenschaft aus Vergleichsgründen zu früheren Erhebungen [2, 3, 7, 12] beschränkte.

Es ließ sich nachweisen, dass die Publikationsaktivitäten der Kliniken einer hohen Streubreite unterworfen waren, dies galt sowohl für die Publikationsanzahl als auch für die generierten IF des einzelnen Mitarbeiters. Die Publikationsaktivität reichte in dem hier analysierten Zeitraum von 16,4 Publikationen pro Mitarbeiter in der bestplatzierten Klinik bis 1,5 Publikationen in der letztplatzierten. Gleiches ergab die Summe der IF: In der nach diesem Maßstab bestplatzierten Klinik erzielte der einzelne Mitarbeiter durchschnittlich kumuliert 42,1 IF verglichen mit 1,7 IF in der letztplatzierten. Wichtiger noch: Unabhängig, wie man den Maßstab ansetzte (Publikationen pro Chirurg, Publikationen pro publizierendem Chirurg, IF pro Chirurg und IF pro publizierendem Chirurg), die hoch signifikanten Unterschiede zwischen den Klinikquantilen bleiben bestehen und lassen sich nicht wegdiskutieren (Abb. 1). Das Ergebnis entspricht damit einer Untersuchung von Debus et al. [3], die für die deutsche universitäre Herzchirurgie ähnlich große Unterschiede in der Publikationsaktivität mit identischer Methodik nachweisen konnten.

Unterschiedliche Publikationsaktivitäten in der orthopädischen Chirurgie sind auch von anderen berichtet worden. Hohmann et al. [8] untersuchten die Publikationsaktivitäten aller orthopädischen Einrichtungen der USA, gegliedert nach Bundesstaat und Stadt, in 15 hochrangigen orthopädischen Journalen für die Jahre 2010 bis 2014. Die vier Städte New York, Philadelphia, Boston und Chicago waren die aktivsten und für 28 % des akademischen Outputs verantwortlich. Zelle et al. [16] verglichen die Leitungspositionen in universitären und nichtuniversitären orthopädisch-chirurgischen Einrichtungen in den USA und sahen in den Universitäten eine deutlich höhere Publikationsaktivität, wobei sie wie hier Erst- und Letztautorenschaften als Maßstab nahmen. Mit der Übernahme der Spitzenposition ging die Publikationsaktivität der jeweiligen Leiter als Erstautor zurück, was in der vorliegenden Untersuchung nicht geprüft wurde, aber vermutet werden kann: Bei den Erstautorenschaften machten die Professoren nur 34 % der Autoren aus, bei den Letztautorenschaften aber 69,6 %.

Einzelne Departments haben Zelle et al. [16] aber nicht verglichen. Gleiches gilt für die Untersuchung von Ence et al. [4], die die Publikationsaktivitäten in 142 akademischen orthopädisch-chirurgischen Einrichtungen der USA mittels des h‑Index untersuchten. Insgesamt gingen 4663 Chirurgen in die Analyse ein. Das Ergebnis war wenig überraschend: Mit längerer Karrieredauer und höherer akademischer Position stieg der h‑Index des einzelnen Chirurgen an. Entsprechend wiesen auch in der vorliegenden Untersuchung die Professoren durchschnittlich pro Mitarbeiter die höchste Publikationsanzahl und kumulierten IF auf. Was das regionale Benchmarking anging, so beobachteten Ence et al. [4] im Süden der USA verglichen mit den anderen Regionen ein niedrigeren h‑Index, auch waren die großen Einrichtungen aktiver als die kleineren. Dies war in der vorliegenden Untersuchung nicht der Fall; größere Abteilungen mit höherer Anzahl an Mitarbeitern waren nicht aktiver als kleinere, was die Anzahl der Publikationen und die kumulierten IF pro Mitarbeiter anging (Abb. 2).

Insgesamt ist bisher ein umfassendes Benchmarking aller unfallchirurgisch/orthopädischen Universitätskliniken eines Landes nicht veröffentlicht worden.

Die Frage ist, worauf die erheblichen Unterschiede, die hier zwischen den Kliniken gefunden wurden, zurückzuführen sind. Nach Gebhard et al. [5] kann es allein an den zugewiesenen Geldmitteln nicht liegen, auch scheidet die Zahl der Mitarbeiter per se aus, da das Benchmarking in der vorliegenden Form sich auf den einzelnen Mitarbeiter bezieht und nicht auf die Gesamtzahl der Publikationen einer Klinik. Schwarzer et al. [13] fanden bei einem Vergleich von Allgemeinchirurgie, Kardiologie und Herzchirurgie eine eindeutige Beziehung zwischen der Zahl der Mitarbeiter und der Zahl der Publikationen einer Disziplin, ein zu erwartendes Ergebnis. Umgerechnet auf den einzelnen Mitarbeiter waren die Unterschiede zwischen den Disziplinen aber nicht mehr signifikant. Sie schlossen daraus, dass in allen drei Disziplinen für den einzelnen Mitarbeiter die gleiche Zeit für Publikationsaktivitäten zur Verfügung steht – oder genutzt wird.

In einer Befragung zur Vereinbarkeit von klinischer und akademischer Entwicklung an orthopädischen und unfallchirurgischen Universitätskliniken in Deutschland erhielten Bernstein et al. [1] die Antwort, dass strukturelle (z. B. Drei-Viertel-Forschungsstelle) Voraussetzungen und leistungsbezogene bzw. auch eigenständige Einteilung zur Freistellung im Rahmen einer insgesamt transparenten Planbarkeit von Weiterbildung und beruflicher Entwicklung wesentliche Voraussetzungen für den Erhalt von Forschungsmotivation seien. Es ist demnach gut vorstellbar, dass die unterschiedlichen Publikationsaktivitäten in den einzelnen Kliniken auf unterschiedlichen Rahmenbedingungen und unterschiedlicher Motivation beruhen. Dabei ist es offensichtlich nicht allein die Zeit, die für wissenschaftliche Betätigung zur Verfügung steht, sondern auch ein unterschiedliches wissenschaftliches Klima. Nur dies kann erklären, warum vergleichbar große Unterschiede auch für andere Fächer beobachtet wurde [3, 7, 11, 12]. Granger et al. [6] haben dies an dem Beispiel einer einzelnen Klinik zeigen können: Mit Einführung wissenschaftlicher Koordinatoren, eines wissenschaftlichen Kurrikulums, der Etablierung von Forschungsteams und einer zentralisierten Forschungsdatenbasis stieg die wissenschaftliche Produktivität signifikant an.

Andererseits demonstriert eine Untersuchung von Johnson et al. [9] die Wichtigkeit von verfügbarer Zeit für Publikationsaktivitäten. Sie verglichen die Publikationsaktivitäten von 10 orthopädisch/chirurgischen Weiterbildungsprogrammen vor und nach Einführung des Arbeitszeitgesetzes in den USA. Die Autoren spekulierten, dass mit gesetzlicher Beschränkung der Dienstzeiten die wissenschaftlichen Aktivitäten ansteigen würden. Verglichen wurden die Jahre 2008 bis 2011 mit den Jahren 2012 bis 2015. Nach Korrektur der Daten hinsichtlich der Zahl der Facharztanwärter (Residents) pro Jahr ließ sich tatsächlich eine signifikante Steigerung in den Publikationsaktivitäten der Residents nachweisen. Allerdings gab es keinen signifikanten Unterschied in den finanziell unterstützten Studien oder in der Anzahl an Publikationen, bei denen Residents Erstautoren waren.

Williams et al. [15] haben die wissenschaftlichen Aktivitäten von 125 Weiterbildungsprogrammen in der orthopädischen Chirurgie der USA mit insgesamt 1690 Residents untersucht. Sie fanden eine signifikante Beziehung zwischen der im Programm vorgesehen Zeit für Forschungsaktivitäten und der Zahl der Erstautorenschaften der Residents. Residents, die über die gesamte Ausbildungszeit hinweg einen Zeitrahmen für Forschung zur Verfügung hatten, waren signifikant aktiver als solche mit einer Blockzeit (erst recht galt das für die Programme, in denen gar keine Zeit für Forschungsaktivitäten festgelegt war). Die Autoren betonten, dass es neben dem Zeitrahmen auf die Struktur der Forschungsaktivitäten, das wissenschaftliche Mentoring angehender Orthopäden/Unfallchirurgen und auch die finanzielle Unterstützung von Forschungsprogrammen ankomme. Letztere Einflüsse seien aber bisher nicht dezidiert untersucht worden.

Krueger et al. [10] haben diese Aussage indirekt bestätigt. Bei einem Vergleich der Publikationsaktivitäten von drei orthopädischen Weiterbildungsprogrammen fanden sie keine signifikanten Unterschiede zwischen Einheiten, die eine feste Zeit für Residents zur Forschung zur Verfügung stellten, und solchen, in denen dies nicht der Fall war. Sie meinten, dass die Forschungsaktivitäten eines Programms (gemessen an Zahl und Qualität der Publikationen) mehr von dem Engagement und dem Forschungsinteresse der Leitung und ihren Mentoreigenschaften abhängen. Nach diesen Autoren kommt es vor allem darauf an, sich auf eine motivierte forschungsaktive Fakultät und ihre Pflege zu fokussieren.

### Limitationen

Einschränkend muss zugegeben werden, dass in der vorliegenden Untersuchung die Publikationsaktivitäten der Führungsmannschaften der unfallchirurgisch/orthopädischen Kliniken und nicht die der wissenschaftlichen Mitarbeiter insgesamt überprüft wurden, was nicht mit der Publikationsleistung der einzelnen Klinik im 10-Jahres-Zeitraum gleichgesetzt werden darf. Dies ergab sich aus methodischen Gründen, um später – bei identischen Vorgaben – die Publikationsaktivitäten der Führungsmannschaften der universitären Unfallchirurgie/Orthopädie mit denen anderer Fächer – wie Gefäßchirurgie, Herzchirurgie und Viszeralchirurgie – vergleichen zu können.

Andererseits gingen die Publikationsaktivitäten der wissenschaftlichen Mitarbeiter ohne Führungsposition doch in das Benchmarking ein: Bei 3845 von 4438 Publikationen (86,6 %) – alle Doppelpublikationen mit Erst- und Letztautorenschaft der Führungsmannschaft ausgeschlossen – waren entweder der Erst- oder der Letztautor kein Mitglied der aktuellen Führungsmannschaft und wurden so sehr wohl bei den Klinikaktivitäten miterfasst.

Hinzu kommt, dass sich die Untersuchung auf klinisch tätige Mitarbeiter beschränkte, die Aktivitäten reiner Forschungsmitarbeiter wurden nicht registriert. Kliniken, die innerhalb des Beobachtungszeitraums einen Wechsel in der Abteilungsleitung hatten, oder Kliniken, bei denen Oberärzte im Beobachtungszeitraum außeruniversitäre Positionen übernommen hatten, sind möglicherweise in den Publikationszahlen unterrepräsentiert, sofern die in der Position Nachfolgenden weniger veröffentlichten als ihre Vorgänger. Andererseits können aber auch Neubesetzungen aktiver als ihre Vorgänger in den letzten 10 Jahren gewesen sein, ein neuer Chef oder Mitarbeiter brachte ja in die Auswertung seine Publikationen der letzten 10 Jahre ein.

### Schlussbetrachtung

Alle diese Einwände verändern aber nicht das Gesamtbild: Die Publikationsaktivitäten unfallchirurgischer Universitätskliniken in Deutschland zeigen eine erhebliche Spanne, wie sie vor Erstellung dieser Analyse nicht erwartet worden war. Sie können bei den gravierenden Unterschieden auch nicht allein mit den Rahmenbedingungen erklärt werden und haben vielmehr auch etwas mit unterschiedlicher Forschungsmotivation zu tun. Die Gründe hierfür sollten weiter untersucht werden.
